# *In Vivo* Antimalarial Activity of the 80% Methanolic Root Bark Extract and Solvent Fractions of* Gardenia ternifolia* Schumach. & Thonn. (Rubiaceae) against* Plasmodium berghei*

**DOI:** 10.1155/2018/9217835

**Published:** 2018-06-12

**Authors:** Dejen Nureye, Solomon Assefa, Teshome Nedi, Ephrem Engidawork

**Affiliations:** ^1^Department of Pharmacy, Mizan-Tepi University, P.O. Box 260, Southwest Ethiopia, Ethiopia; ^2^Department of Pharmacology and Clinical Pharmacy, College of Health Sciences, School of Pharmacy, Addis Ababa University, P.O. Box 1176, Addis Ababa, Ethiopia

## Abstract

**Background:**

Evolution of antimalarial drug resistance makes the development of new drugs a necessity. Important source in search of such drugs is medicinal plants.* Gardenia ternifolia *plant is used in Ethiopian traditional medicine for the treatment of malaria and is endowed with* in vitro *antimalarial activity. Herein, the* in vivo* antimalarial activity of the plant was investigated.

**Methods:**

Acute toxicity was carried out using a standard procedure. A 4-day suppressive test was employed to evaluate the antimalarial effect of methanolic crude extract and solvent fractions of the plant. The curative and prophylactic effect of crude extract was further tested by Ranes's test and residual infection procedure, respectively, using* Plasmodium berghei *(ANKA strain) in Swiss albino mice.

**Results:**

The chemosuppressive effect exerted by the crude extract and fractions ranged between 30-59% and 14-51%, respectively. Curative and prophylactic effects of the crude extract were in the range of 36-63% and 24-37%, respectively. All dose levels of the crude extract prevented loss of weight, reduction in temperature, and anemia on early and established infection. Butanol and chloroform fractions also did reverse reduction in temperature, body weight, and packed cell volume.

**Conclusions:**

The results indicated that the plant has a promising antiplasmodial activity and it could be considered as a potential source to develop new antimalarial agents.

## 1. Background

Malaria continues to remain an important cause of illness and death in countries in which it is endemic [1]. In 2016, there were an estimated 212 million cases of malaria globally and led to 445, 000 deaths, most of which were in African children under the age of 5. Irrespective of the effort made to minimize malaria incidence and prevalence globally, the burden of morbidity and mortality is high in African Region [2]. Despite a decreased malaria mortality rate in Ethiopia by > 40% since 2010, high prevalence was observed in contrast to high household coverage of control interventions [2, 3].

The evolution of strains resistant to commonly used antimalarial drugs and the lack of inexpensive new drugs jeopardized the fight against malaria. This triggers the continuing need of research for novel antimalarial compounds [4, 5]. History has taught us how traditional medicines like* Artemisia annua* L. and bark of* Cinchona pubescens *Vahl are valuable in discovering new antimalarial drugs [6–8].

About 90% of the Ethiopian populations are dependent on traditional medicines for the management of diseases in both humans and animals [9]. The widespread use of traditional medicines in Ethiopia could be attributed to cultural acceptability, physical accessibility, and economic affordability as compared to modern medicine [10]. There are a number of plants utilized in Ethiopian ethnomedicine practice for the treatment of malaria. Studies conducted on several traditionally confirmed antimalarial activities of Ethiopian medicinal plants, such as* Calpurnia aurea *[11]*, Croton macrostachyus *[12],* Asparagus africanus *[13],* Withania somnifera *[14],* Dodonaea angustifolia* [15], and* Phytolacca dodecandra* [16].

*Gardenia ternifolia *Schumach. & Thonn. (Rubiaceae) is a shrub or small tree about 5 to 10 m high [17, 18]. In Ethiopia, stem and root barks of* G. ternifolia* are taken as a remedy for malaria [19, 20]. In addition, the plant is used medicinally by tribal healers to treat hemorrhoid lesions [21], gum bleeding [22], and stomachache [23] in human and ulcerative lymphangitis in livestock [24].

The leaves exudates of the plant showed* in vitro* antiplasmodial activity against* Plasmodium falciparum* [25]. Moreover, fruit extract of the plant possesses* in vitro* antiparasitic activity against* Theileria lestoquardi *that infects ruminant erythrocytes [26, 27]. Thus, based on the claims made by the traditional healers and* in vitro *antimalarial activity of the leaves surface exudates, the present study evaluated the* in vivo* antimalarial activity of 80% methanol extract and solvent fractions of the root barks of* G. ternifolia*.

## 2. Methods

### 2.1. Plant Collection

Root barks of* G. ternifolia *were collected in October, 2015, from its natural habitat around Mizan-Teferi, 581 km South West of Addis Ababa. The fresh root barks were wrapped with plastic sheets during transportation. The collected plant was identified as* G. ternifolia* by a taxonomist at the National Herbarium, College of Natural and Computational Sciences, Addis Ababa University, where a voucher specimen (no. DN 001) was deposited for a future reference.

### 2.2. Plant Extraction and Fractionation

Fresh root barks of the plant were thoroughly washed with distilled water to remove dirt and soil. The root bark samples were then air-dried under shade and pulverized into powder using sterile pestle and mortar. The powder (200 g) was cold macerated with 600 mL of 80% methanol in an Erlenmeyer flask for three consecutive days at room temperature. The same volume of solvent was used for successive extraction of the residues. The extraction process was facilitated using a mechanical shaker at 120 rpm. The extract (19% w/w) was transferred into vials and kept at −20°C until use.

The crude hydroalcoholic extract was subjected to fractionation using solvents with differing polarity (chloroform, n-butanol, and water). For fractionation purpose, crude extract was suspended in a separatory funnel using distilled water and the suspension was shaken with chloroform and n-butanol successively. All the fractions were kept in an amber glass bottle and stored in a refrigerator (−20°C). The percentage yield of butanol, chloroform, and aqueous fractions was 28.21%, 26.27%, and 45.52%, respectively.

### 2.3. Preliminary Phytochemical Screening

Both crude extract and solvent fractions were screened for the presence and absence of different phytochemical constituents to relate the secondary metabolites with antimalarial activity. Hence, tests for alkaloids, anthocyanins, flavonoids, glycosides, phenolic compounds, saponins, steroidal compounds, tannins, and terpenoids were carried out following standard procedures described by Debella (2002) [28] and Godgate and Rajaram (2013) [29].

### 2.4. Experimental Animals and Parasite

Healthy Swiss albino mice (22-31 g), aged 4-6 weeks, were purchased from Ethiopian Public Health Institute (EPHI). Mice were maintained in the laboratory under standard condition (temperature of 22 ± 3°C, relative humidity of 40-50% and 12 h light/12 h dark cycle) with a commercial food and water* ad libitum.* Mice were acclimatized for one week before the study. All procedures and techniques used in this study were in accordance with the guide for care and use of laboratory animals [30].

Chloroquine sensitive strain of* P. berghei* (ANKA ) was obtained from EPHI. The parasites were maintained by serial passage of blood from infected mice to noninfected ones on weekly basis.

### 2.5. Acute Toxicity Study

Noninfected female Swiss albino mice, fasted overnight, were used for the acute toxicity study. Following the period of fasting, the animals were weighed and 2000 mg/kg of the crude extract was administered by oral gavage. Food was then withheld for further 1 to 2 h. First, one female mouse was dosed and the mouse was observed continuously for the first 30 min and intermittently for 4 h, over a period of 24 h. Since no death was observed, another four female mice were dosed and gross behavioral changes like loss of appetite, hair erection, lacrimation, tremors, convulsions, mortality and other signs of toxic manifestation were observed for 14 days after administration of the extract [31].

### 2.6. *In Vivo* Antimalarial Screening

#### 2.6.1. Parasite Inoculation

Albino mice previously infected with* P. berghei* having different levels of parasitemia (30-37%) were used as donors [32]. Donor mice were placed in closed chamber and euthanized with inhalation of anesthesia gas and infected blood was collected by cardiac puncture into heparinized vacutainer tube containing 0.5% trisodium citrate. The blood was then diluted in normal saline (0.9%) based on parasitemia level of the donor mice and the red blood cell (RBC) count of normal mice so that the final suspension would contain about 1×10^7^ parasitized red blood cells (PRBCs) in every 0.2 mL suspension [33]. Each mouse used in the study was infected intraperitoneally with 0.2 mL infected blood containing about 1×10^7^* P. berghei* parasitized RBCs.

#### 2.6.2. Grouping and Dosing of Animals

To evaluate the antimalarial activity of crude extract and solvent fractions, infected mice were randomly divided into five groups of 6 mice each. For each test, three groups (I-III) were treated with the crude extract or solvent fractions at 200 mg/kg, 400 mg/kg, and 600 mg/kg, respectively. The remaining two groups served as positive and negative controls and received chloroquine 25 mg/kg (CQ25) and 10 mL/kg of vehicle, respectively. Each dose was reconstituted by distilled water and administered orally. Duration of administration depended on the type of test performed.

#### 2.6.3. The 4-Day Suppressive Test

Evaluation of schizonticidal activity of the root barks of* G. ternifolia* crude extract and solvent fractions on early infection was carried out according to the method described by Peter et al. [34]. Treatment was started on the first day (D0) and 2 h postinfection and continued daily for 4 days (D1-D3). On the 5^th^ day (D4), blood was collected from the tail of each mouse using clean, nongreasy slides and thin films were made accordingly to determine parasitemia and percentage inhibition. In addition, each mouse was observed daily for determination of survival time.

#### 2.6.4. Rane's (Curative) Test

Method described by Ryley and Peters [35] was employed to evaluate the curative potential of the plant. Thirty male mice were intraperitoneally inoculated with standard inoculums of 1×10^7^* P. berghei* PRBCs on the first day (Day 0). Seventy-two hours later, animals were randomly divided into 5 groups and dosed accordingly. Treatment continued once daily until the 7^th^ day (D3-D6). Geimsa stained thin blood film was prepared from the tail of each mouse daily for 5 days to monitor parasitemia level. Survival time was determined over a period of 30 days (D0-D29).

#### 2.6.5. Prophylactic Test

The prophylactic activity of the extract was tested using the residual infection procedure described by Peters [36]. Adult male mice were weighed, randomized into five groups (n=6), and treated for four consecutive days. On the 5^th^ day (D4), all mice were infected with the* Plasmodium*. Seventy-two hour postinfection, parasitemia level, weight change, temperature, and packed cell volume (PCV) were determined. Then after, the mice were followed for 28 days for their survival to calculate mean survival time.

#### 2.6.6. Packed Cell Volume Measurement

To determine the effectiveness of the extract and fractions in preventing the hemolytic effect of the parasite, PCV was measured [14]. Heparinized capillary tubes were used for the collection of blood from tail of the mice. The capillary tubes were filled to 3/4^th^ of their height with blood and sealed with sealing clay at their dry end. The tubes were then placed on a microhematocrit centrifuge (Centurion Scientific, UK) with the sealed end facing the periphery and centrifuged at 11,000 rpm for 5 min. Finally, the tubes were taken out of the centrifuge and PCV was determined using the standard hematocrit reader (Hawksley and Sons, England) [37]. PCV was calculated using the relation shown below [14]:

(1) PCV=Volume  of  erythrocytes  in  agiven  volume  of  bloodTotal  blood  volume  examined×100

#### 2.6.7. Parasitemia Measurement

The percentage parasitemia was obtained by counting the number of infected RBC out of the total RBC in random microscopic fields. Two stained slides for each mouse were examined [12]. Six different fields on each slide were counted to determine percentage parasitemia using the following formula [38]:

(2) $$\begin{array}{c} \%\;\;parasitemia=\frac{\text{Number of PRBC}}{\text{Total number of RBC}}\times 100 \end{array}$$

Percent parasitemia suppression of the extract and fractions was compared with respect to the controls. Percent parasitemia suppression was calculated using the following formula [38]:

(3) $$\begin{array}{c} \text{Average \% of Parasitemia Suppression}=\frac{\text{Parasitemia in negative control}-\text{Parasitemia in treatment group}}{\text{Parasitemia in negative control}}\times 100 \end{array}$$

### 2.7. Data Analysis

Data were organized, edited, and analyzed using SPSS Version 20. Data obtained from all tests were analyzed with one-way ANOVA followed by Tukey* post hoc* test to compare the levels of parasitemia, survival time, and changes in body weight, PCV, and rectal temperature of the* P. berghei *infected mice between control and treatment groups. Results were deemed statistically significant if P values < 0.05 at 95% confidence intervals.

## 3. Results

### 3.1. Acute Toxicity Study

The acute toxicity study indicated that the extract caused no mortality at dose of 2000 mg/kg. Physical and behavioral observations of the experimental mice also revealed no visible signs of overt toxicity. This suggests that LD_50_ of the extract is greater than 2000 mg/kg.

### 3.2. Four-Day Suppressive Test

#### 3.2.1. Chemosuppressive Effect of the Plant in the Suppressive Test

The 4-day suppressive study revealed that the extract as well as fractions exhibited a significant reduction of parasitemia (p<0.001) compared to the negative control (Table 1). The 600 mg/Kg dose of butanol fraction produced higher parasitemia suppression (51.33%) compared to the same dose of chloroform fraction (40.73%). Of the fractions, aqueous fraction exhibited the lowest chemosuppression against* P. berghei*. Moreover, the mean survival time of mice treated with the crude extract and fractions was increased in a dose-dependent manner (Table 1). The standard drug markedly (p<0.001) reduced parasite count to undetectable level and prolonged survival time of infected mice.

#### 3.2.2. Effect on Body Weight and Rectal Temperature in Suppressive Test

The crude extract appeared to significantly avert loss of weight associated with infection as compared to negative control. As indicated in Table 2, all the three doses of the crude extract were able to significantly (p<0.05 for lower, p<0.001 for middle and highest doses) prevent the decrease in rectal temperature caused by* P. berghei *infection compared to negative control.

#### 3.2.3. Effect on Packed Cell Volume in Suppressive Test

Although the effects were lower than CQ25, crude extract (200 and 400 mg/kg), butanol fraction (200 and 400 mg/kg), and chloroform fraction (600 mg/Kg) treated groups significantly prevented reduction of PCV (p<0.001) compared to the vehicle treated group (Table 3).

### 3.3. Rane's Test

#### 3.3.1. Curative Effect of the Plant in Rane's Test

The crude extract at 400 and 600 mg/kg doses revealed a significant (p<0.001) curative effect compared to vehicle treated group (Table 4). Survival period was altered significantly by the lower (p<0.01), middle (p<0.001), and highest (p<0.001) doses of test sample relative to negative control. However, the curative effect achieved with the extract was lower compared to CQ25.

#### 3.3.2. Effect of Crude Extract on Body Weight and Rectal Temperature in Rane's Test

The crude extract exhibited a dose-dependent prevention of body weight reduction in infected mice (Table 5). Moreover, CQ25 and all dose levels of the crude extract significantly (p<0.001) prevented the reduction in rectal temperature compared to the negative control group.

#### 3.3.3. Effect of Crude Extract on Packed Cell Volume in Rane's Test

**Figure 1:**
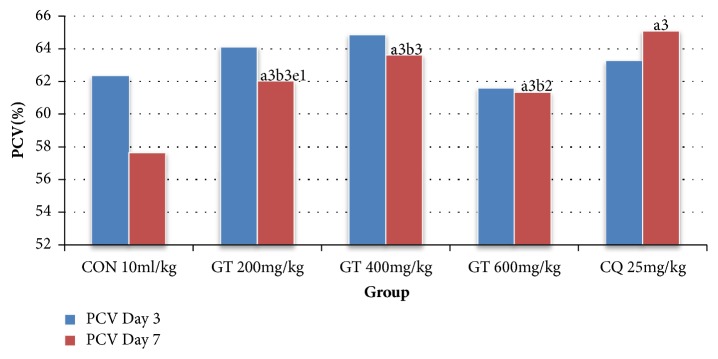
Comparison of packed cell volume in malaria-infected mice treated with crude extract, chloroquine and placebo in the Rane's test. Data are expressed as mean ± SEM (n=6); a, compared to negative control; b, compared to CQ 25 mg/kg; c, compared to 200 mg/kg; d, compared to 400 mg/kg; e, compared to 600 mg/kg; ^1^p< 0.05; ^2^p<0.01, ^3^p<0.001; CON, negative control; CQ, chloroquine (positive control); GT, crude extract.

The crude extract was able to significantly (p<0.001) prevent PCV reduction compared to vehicle treated group (Figure 1).

### 3.4. Prophylactic Effect of the Plant

All doses of the crude extract and CQ25 significantly (p<0.001) suppressed parasite load compared to negative control (Table 6). Although complete eradication was not achieved, maximum suppression (84.83%; p<0.001) of parasitemia was noted by the positive control. Survival time of the infected mice pretreated with the crude extract indicated that only the highest dose was capable of significantly (p<0.01) prolonging survival time compared to control (Table 6).

#### 3.4.1. Effect of Crude Extract on Body Weight and Rectal Temperature in Prophylactic Test

CQ25 and highest dose of extract showed a significant (p<0.001 for CQ25 and p<0.01 for crude extract) protective effect in body weight reduction compared to vehicle treated group (Table 7). Chloroquine treated group considerably prevented weight reduction in comparison to the middle (p<0.01) and lower (p<0.001) doses of crude extract. However, the highest dose of the crude extract appreciably (p<0.001) prevented rectal temperature reduction compared to control mice.

#### 3.4.2. Effect of Crude Extract on Packed Cell Volume in Prophylactic Test

**Figure 2:**
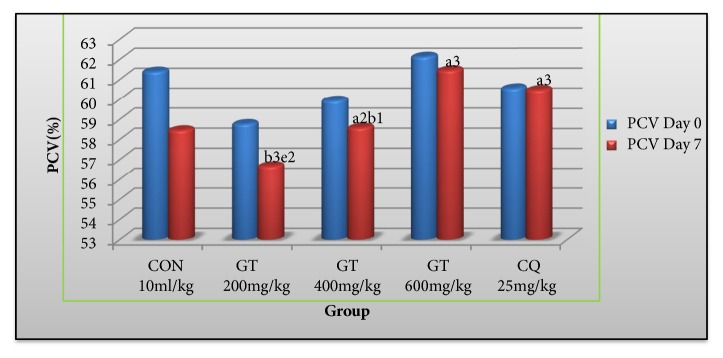
Results on packed cell volume of* Plasmodium berghei* infected mice treated with the 80% methanolic extract of* Gardenia ternifolia* root barks in the prophylactic test. Data are expressed as mean ± SEM (n=6); a, compared to CON; b, compared to CQ 25 mg/kg; c, compared to 200 mg/kg; d, compared to 400 mg/kg; e, compared to 600 mg/kg; ^1^p< 0.05; ^2^p<0.01, ^3^p<0.001, D_0_ = preinoculated value on day three, D_7_ = postinoculated value on day seven.

Multiple comparison among PCV reductions (Figure 2) indicated that the highest dose (p<0.001) and middle dose (p<0.01) of crude extract and the standard drug (p <0.001) were able to significantly attenuate PCV reduction compared to the negative control.

### 3.5. Preliminary Phytochemical Screening

Phytochemical screening revealed the presence of tannins, alkaloids, anthocyanins, terpenoids, saponins, phenols, steroids, and flavonoids in crude extract of* G. ternifolia* root barks. Of all the fractions, butanol fraction exhibited a maximal number of secondary metabolites (Table 8).

## 4. Discussion

Death or signs of toxicity was not observed in oral acute toxicity evaluation of the test extract and this could explain the safe folkloric use of the plant.* In vivo* model was opted for this study because it takes into account any prodrug effect and the likelihood of immune system in controlling infection as compared to* in vitro* study [33].

In the 4-day suppressive test, parasite suppression exhibited by the extract was comparable with other study done on* Aloe debrana *[32]. Moreover, the crude extract prolonged survival time in early parasite infection, which is concordant with study conducted on ethanolic leaf extract of* Chromolaena odorata* [39].

Among the fractions, butanol and chloroform fractions exerted a better chemosuppressive effect than aqueous fraction, suggesting the possible localization of active ingredients in these two fractions. This finding is in accordance with other studies in which butanol fraction exhibited a superior antimalarial activity [40, 41]. This effect could be attributed to the existence of alkaloids, flavonoids, saponins, and terpenoids in butanol fraction. On the other hand, aqueous fraction produced the lowest inhibition of parasitemia in the 4-day suppressive test. This could probably emanate from the absence of most of the bioactive secondary metabolites from this fraction. The finding is in line with other studies in which the antimalarial effect of aqueous fraction is less than chloroform and butanol fractions [12].

The chemosuppressive effect of the plant is similar to other species of the same genus such as* Gardenia sokotensis* [42] and* Gardenia Lutea* [43]. Previous study also conveyed that flavonoids and steroids isolated from* G. ternifolia* leaf surface exudates showed an* in vitro* antiplasmodial activity (IC_50_ values 1.06 and 0.94 *μ*g/mL) against* falciparum* strains [25].

Body weight loss, reduction in PCV, and low body temperature are cardinal signs of malaria-infected mice [44]. Hence, ideal plant extracts with antimalarial activity are expected to prevent malaria-associated reduction of body weight, PCV, and temperature due to the rise in parasitemia.

The highest doses of butanol and chloroform fractions showed a remarkable increment in body weight compared to the infected but untreated mice. This activity might have been resulted from the overall improvement in PCV, rectal temperature, and parasite clearance among treated mice [45]. However, aqueous fraction did not prevent weight reduction. This finding is in agreement with previous studies on aqueous fraction of other plants [46, 47].

A decrease in the metabolic rate of* P. berghei* infected mice occurs before death and is accompanied by a corresponding decrease in body temperature [15]. Active compound(s) should prevent the rapid dropping of rectal temperature. In 4-day suppressive test, all doses of the crude extract and middle and larger doses of butanol and chloroform fractions protected the decrease in rectal temperature associated with infection. Overall, this activity might probably indicate the ability of plant to ameliorate some pathological processes of malaria that cause reduction in body temperature [15].

In the chemosuppressive study, the extract and fractions of* G. ternifolia* significantly prevented PCV reduction in a dose-dependent manner. This effect is in line with the RBC protection effect of crude extract of* Clerodendrum myricoides* leaves,* Dodonaea angustifolia* seeds [32, 46], and* Croton macrostachyus* leaves [12].

In established infection, the crude extract exerted significant suppression of parasitemia. As indicated in the results section, all treated groups brought about reduction of parasitemia after the second dose; however, the standard drug started its activity right after the first dose. This delay of activity may be indicative of the need for a loading dose or the extract might have a slower onset of action compared to chloroquine. Since it is desirable to have both suppressive and curative activities in a phytodrug, it may be possible to consider this plant as a potential source of antimalarial agents [48].

The chemosuppresive effect of extract on established infection was higher than the 4-day suppressive test. This pronounced antimalarial activity observed in the established infection test may be due to inhibitory effect of the extract on generation of free radicals and hemolytic principles resulting from high parasitaemia level [49, 50]. This is further supported by the* in vitro* analysis in which flavonoid aglycones from the leaves of* G. ternifolia* exhibiting antioxidant activity that can counteract the oxidative damage induced by the malaria parasite [19].

To be active, the sample should suppress percent parasitemia by ≥ 30% [51]. Based on this assertion, the crude extract, butanol, and chloroform fractions are active against malaria infection.

## 5. Conclusion

The present study indicated that crude extract and solvent fractions of* G. ternifolia *possess a promising antimalarial activity, with higher effect exhibited by the crude extract. The antimalarial action of the extract and solvent fractions has been attributed to the presence of semipolar to nonpolar ingredients in the root barks of the plant. Therefore, the extracts and solvent fractions of* G. ternifolia* could potentially be used as a new source for the development of new plant-based antimalarial agent.

## Figures and Tables

**Table 1:** Antimalarial activity of 80% methanol extract and solvent fractions of *Gardenia ternifolia* root barks against *Plasmodium berghei* in 4-day suppressive test.

Group	% Parasitemia	% Suppression	Survival Time
CON	53.95±0. 71	0.00	6.33±0.21
GT200	36.38±0.83	32.58^a3b3d3e3^	8.50±0.43^a2b3d3e3^
GT400	28.58±0.65	47.02^a3b3e3^	11.17±0.31^a3b3e3^
GT600	21.98±0.72	59.25^a3b3^	13.50±0.43^a3b3^
CQ25	0.00±0.00	100.00^a3^	28.00±0.00^a3^

CON	48.64±0.94	0.00	6.50±0.22
BF200	33.61±0.90	30.89^a3b3d3e3^	8.83±0.60^a1b3e3^
BF400	27.93±0.51	42.45^a3b3e3^	10.50±0.56^a3b3e1^
BF600	23.68±0.59	51.33^a3b3^	12.83±0.60^a3b3^
CQ25	0.00±0.00	100.00^a3^	28.00±0.00^a3^

CON	50.49±0.60	0.00	6.33±0.21
CF200	38.12±0.68	24.51^a3b3d3e3^	7.17±0.31^b3e3^
CF400	34.77±0.63	31.13^a3b3e3^	8.00±0.45^a1b3e2^
CF600	29.93±0.35	40.73^a3b3^	10.00±0.45^a3b3^
CQ25	0.00±0.00	100.00^a3^	28.00±0.00^a3^

CON	46.86±1.20	0.00	6.67±0.33
AF200	39.99±1.19	14.67^a3b3e3^	7.00±0.37^b3^
AF400	37.92±0.57	19.09^a3b3e1^	7.50±0.22^b3^
AF600	34.80±0.68	25.75^a3b3^	7.83±0.40^b3^
CQ25	0.00±0.00	100.00^a3^	28.00±0.00^a3^

Data are expressed as mean ± SEM (n=6); a, compared to CON; b, to CQ 25; c, to 200 mg/kg; d, to 400 mg/kg; e, 600 mg/kg: ^1^p<0.05, ^2^p<0.01, ^3^p<0.001; CON, negative control, received distilled water 10 ml/kg; GT = crude extract of *Gardenia ternifolia*, BF = n-butanol fraction, CF = chloroform fraction, AF = aqueous fraction, and CQ = chloroquine; numbers (25,200,400,600) refer to dose in mg/kg.

**Table 2:** Body weight and rectal temperature change of infected mice treated with crude extract and solvent fractions of *Gardenia ternifolia* root barks in 4-day suppressive test.

Group	Weight (gram)			Temperature (^0^c)		
	D_0_	D_4_	% Change	D_0_	D_4_	% Change
CON	25.76±1.17	21.65±0.94	-13.55	35.85±0.38	33.15±0.33	-7.51
GT200	26.78±1.34	24.73±1.41	-7.80^a1b3e1^	36.65±0.28	35.10±0.15	-4.21^a1b3e2^
GT400	27.37±1.01	26.37±1.16	-3.67^a2b3^	36.50±0.25	35.58±0.17	-2.50^a3b2^
GT600	28.51±0.56	28.46±0.81	-0.20^a3b2^	36.60±0.23	36.30±0.48	-0.81^a3^
CQ25	28.02±1.11	30.95±1.10	10.55^a3^	36.32±0.34	36.82±0.25	1.40^a3^

CON	27.78±0.49	24.04±0.53	-11.14	36.75±0.31	34.25±0.26	-6.80
BF200	28.91±0.48	26.81±0.42	-7.25^b3e1^	37.35±0.21	35.53±0.16	-4.85^b3e1^
BF400	29.24±0.31	28.09±0.24	-3.91^a2b3^	37.45±0.21	36.02±0.30	-3.83^a2b3^
BF600	27.93±0.48	27.77±0.60	-0.61^a3b3^	36.90±0.24	36.00±0.33	-2.45^a3b3^
CQ25	27.07±0.70	30.23±0.99	11.60^a3^	36.80±0.43	37.45±0.21	1.80^a3^

CON	26.33±1.15	22.44±0.97	-12.20	36.30±0.53	33.55±0.33	-7.54
CF200	26.85±0.98	24.15±1.24	-10.29^b3^	36.53±0.40	34.30±0.48	-6.12^b3^
CF400	27.60±0.62	25.43±0.67	-7.91^b3^	36.70±0.33	35.05±0.43	-4.51^a1b3^
CF600	28.41±0.63	27.20±0.60	-4.27^a3b3^	36.80±0.41	35.55±0.40	-3.39^a2b3^
CQ25	28.10±0.82	31.39±0.76	11.83^a3^	36.70±0.40	37.28±0.26	1.65^a3^

CON	26.93±0.73	23.65±0.90	-10.72	36.45±0.25	33.10±0.50	-9.21
AF200	27.74±0.85	25.04±0.91	-9.82^b3^	36.90±0.31	33.80±0.34	-8.41^b3^
AF400	26.92±0.79	24.60±0.76	-8.61^b3^	36.35±0.36	33.70±0.34	-7.29^b3^
AF600	28.61±0.61	26.34±0.65	-7.96^b3^	37.08±0.40	34.52±0.43	-6.92^b3^
CQ25	28.32±0.63	31.21±0.68	10.22^a3^	37.00±0.35	37.90±0.23	2.45^a3^

Data are expressed as mean ± SEM (n=6); a, compared to CON; b, compared to CQ 25 mg/kg; c, compared to 200 mg/kg; d, compared to 400 mg/kg; e, compared to 600 mg/kg; ^1^p< 0.05; ^2^p<0.01, ^3^p<0.001; CON, negative control; GT = crude extract, BF = n-butanol fraction, CF = chloroform fraction, AF = aqueous fraction, and CQ = chloroquine; D_0_ = pretreatment value on day zero, D_4_ = posttreatment value on day four; numbers (25,200,400,600) refer to dose in mg/kg.

**Table 3:** Effects of crude extract and fractions of *Gardenia ternifolia* root barks on packed cell volume of infected mice in 4-day suppressive test.

Group	Packed Cell Volume		
	D_0_	D_4_	% change
CON	59.91±2.51	55.11±2.48	-7.99
GT200	64.86±1.79	62.57±1.90	-3.56^a2b3e1^
GT400	61.98±2.40	61.22±2.63	-1.27^a3b2^
GT600	56.96±1.43	57.00±1.40	0.09^a3b1^
CQ25	59.77±1.75	62.19±1.89	4.05^a3^

CON	63.68±2.72	59.11±2.73	-7.23
BF200	63.10±1.16	60.58±1.07	-3.97^a1b3e2^
BF400	61.09±1.95	60.11±1.99	-1.58^a3b3^
BF600	64.02±2.96	63.91±2.94	-0.17^a3b3^
CQ25	65.21±1.68	68.23±1.72	4.64^a3^

CON	65.15±1.91	60.26±1.81	-7.51
CF200	62.11±1.44	58.05±1.02	-6.47^b3e3^
CF400	58.97±1.52	56.62±1.47	-3.97^a2b3^
CF600	62.13±1.67	60.95±1.77	-1.93^a3b3^
CQ25	59.82±1.74	62.57±2.15	4.52^a3^

CON	64.14±3.90	59.55±3.78	-7.22
AF200	62.66±2.50	58.41±2.29	-6.77^b3^
AF400	64.71±2.30	60.86±2.07	-5.91^b3^
AF600	58.71±1.29	55.50±1.25	-5.46^b3^
CQ25	60.48±2.60	63.24±2.44	4.65^a3^

Data are expressed as mean ± SEM (n=6); a, compared to negative control; b, compared to CQ 25 mg/kg; c, compared to 200 mg/kg; d, compared to 400 mg/kg; e, compared to 600 mg/kg; ^1^p< 0.05; ^2^p<0.01, ^3^p<0.001; CON, negative control (received 10 ml/kg distilled water); CQ, chloroquine; GT, *G. ternifolia*; D_0_ = pretreatment value on day zero, D_4_ = posttreatment value on day four; numbers (25,200,400,600) refer to dose in mg/kg.

**Table 4:** Antimalarial activity of the hydroalcoholic extract of *Gardenia ternifolia* root barks against *Plasmodium berghei* in Rane's test.

Group	% Parasitemia (D7)	% Inhibition	Survival Time
CON	38.12±0.75	0.00	7.17±0.17
GT200	24.29±0.39	36.29^a3b3d3e3^	9.00±0.26^a2b3d3e3^
GT400	18.26±0.61	52.11^a3b3e3^	11.50±0.43^a3b3e3^
GT600	14.02±0.81	63.22^a3b3^	14.00±0.58^a3b3^
CQ25	0.00±0.00	100.00^a3^	28.00±0.00^a3^

Data are expressed as mean ± SEM (n=6); a, compared to CON; b, compared to CQ 25 mg/kg; c, compared to 200 mg/kg; d, compared to 400 mg/kg; e, compared to 600 mg/kg; ^1^p< 0.05; ^2^p<0.01, ^3^p<0.001; CON, negative control (received 10 ml/kg distilled water); CQ, chloroquine; GT = crude extract of *G. ternifolia*. Numbers (25,200,400,600) refer to dose in mg/kg.

**Table 5:** Body weight and rectal temperature of malaria infected mice before and after treatment with 80% methanolic extract of root barks of *Gardenia ternifolia* in Rane's test.

Group	Weight (gram)			Temperature (^0^c)		
Dose/kg	D_3_	D_7_	% Change	D_3_	D_7_	% Change
CON	25.64±1.20	22.19±1.15	-13.54	36.40±0.25	33.10±0.26	-9.05
GT200	24.95±1.14	23.19±0.95	-6.90^a2b3e2^	35.90±0.22	34.45±0.20	-4.04^a3b3e3^
GT400	25.56±1.07	24.82±1.22	-3.02^a3b3^	35.87±0.24	35.10±0.35	-2.14^a3b3^
GT600	26.76±0.82	26.72±0.69	-0.09^a3b3^	36.00±0.23	35.75±0.25	-0.69^a3b2^
CQ25	25.79±1.04	28.85±1.22	11.86^a3^	36.07±0.25	36.85±0.17	2.18^a3^

Data are expressed as mean ± SEM (n=6); a, compared to CON; b, compared to CQ 25 mg/kg; c, compared to 200 mg/kg; d, compared to 400 mg/kg; e, compared to 600 mg/kg; ^1^p< 0.05; ^2^p<0.01, ^3^p<0.001; CON, negative control; CQ, chloroquine (positive control); GT, Crude extract; D_3_ = pre-Rx value on day three, D_7_ = post-Rx value on day seven; numbers (25,200,400,600) refer to dose in mg/kg.

**Table 6:** Parasitemia, percentage suppression and survival time of malaria infected mice treated with crude extract of *Gardenia ternifolia* root barks in repository test.

Group	% Parasitemia	% Suppression	Survival Time
CON	21.49±0.70	0.00	6.67±0.21
GT200	16.24±0.57	24.43^a3b3e3^	7.33±0.21^b3e1^
GT400	15.25±0.49	29.04^a3b3e1^	8.50±0.84^b3^
GT600	13.36±0.33	37.83^a3b3^	9.50±0.56^a2b3^
CQ25	3.26±0.38	84.83^a3^	16.00±0.77^a3^

Data are expressed as mean ± SEM (n=6); a, compared to CON; b, compared to CQ 25 mg/kg; c, compared to 200 mg/kg; d, compared to 400 mg/kg; e, compared to 600 mg/kg; ^1^p< 0.05; ^2^p<0.01, ^3^p<0.001; CON, negative control; CQ, chloroquine (positive control); GT, crude extract.

**Table 7:** Body weight and rectal temperature of *Plasmodium berghei* infected mice treated with crude extract of *Gardenia ternifolia* root barks in repository test.

Group	Weight (gram)			Temperature (^0^c)		
	D_0_	D_7_	% Change	D_0_	D_7_	% Change
CON	27.82±0.86	26.13±1.02	-6.19	36.90±0.36	34.98±0.45	-5.20
GT200	27.68±0.93	26.28±0.86	-5.01^b3^	36.75±0.29	35.25±0.29	-4.08^b3e1^
GT400	28.58±0.49	27.42±0.40	-4.02^b2^	37.20±0.22	35.98±0.27	-3.27^b2^
GT600	28.55±0.76	27.88±0.80	-2.36^a2^	36.80±0.24	36.17±0.38	-1.73^a3^
CQ25	28.54±0.64	28.63±0.76	0.28^a3^	37.00±0.31	36.85±0.36	-0.41^a3^

Data are expressed as mean ± SEM (n=6); a, compared to CON; b, compared to CQ 25 mg/kg; c, compared to 200 mg/kg; d, compared to 400 mg/kg; e, compared to 600 mg/kg; ^1^p< 0.05; ^2^p<0.01, ^3^p<0.001; CON, negative control; CQ, chloroquine (positive control); GT, crude extract of *G. ternifolia*; D_0_ = preinoculated value on day three, D_7_ = postinoculated value on day seven; numbers (25,200,400,600) refer to dose in mg/kg.

**Table 8:** Preliminary phytochemical screening of the hydroalcoholic extract and solvent fractions of *Gardenia ternifolia* root barks.

Phytoconstituents	Crude Extract	Butanol Fraction	Chloroform Fraction	Aqueous Fraction
Alkaloids	+	+	+	-
Anthocyanins	+	+	-	+
Flavonoids	+	+	+	-
Glycosides	-	-	-	-
Phenols	+	+	-	-
Saponins	+	+	+	+
Steroids	+	+	+	-
Tannins	+	+	+	-
Terpenoids	+	-	+	-

-, absent; +, present.

## Data Availability

Vouchers and dried leaves used for this study are stored at the herbarium at the Addis Ababa University, College of Natural Sciences. The datasets supporting the conclusion of this study are available from the corresponding author on reasonable request.

## Abbreviations

CQ: Chloroquine
IC_50_: 50% inhibitory concentration
LD_50_: Median lethal dose
PCV: Packed cell volume
PRBC: Parasitized red blood cell.
